# Nut Production in *Bertholletia excelsa* across a Logged Forest Mosaic: Implications for Multiple Forest Use

**DOI:** 10.1371/journal.pone.0135464

**Published:** 2015-08-13

**Authors:** Cara A. Rockwell, Manuel R. Guariguata, Mary Menton, Eriks Arroyo Quispe, Julia Quaedvlieg, Eleanor Warren-Thomas, Harol Fernandez Silva, Edwin Eduardo Jurado Rojas, José Andrés Hideki Kohagura Arrunátegui, Luis Alberto Meza Vega, Olivia Revilla Vera, Roger Quenta Hancco, Jonatan Frank Valera Tito, Betxy Tabita Villarroel Panduro, Juan José Yucra Salas

**Affiliations:** 1 Center for International Forestry Research (CIFOR), Lima, Perú; 2 International Center for Tropical Botany, Department of Earth and Environment, Florida International University (FIU), Miami, FL, United States of America; 3 Solutions and Evidence for Environment and Development (SEED), Oxford, United Kingdom; 4 Universidad Nacional Amazónica de Madre de Dios (UNAMAD), Facultad de Ingeniería Forestal y Medio Ambiente, Puerto Maldonado, Madre de Dios, Perú; 5 Centre for Ecology, Evolution and Conservation, School of Environmental Sciences, University of East Anglia, Norwich, United Kingdom; University of Massachusetts, UNITED STATES

## Abstract

Although many examples of multiple-use forest management may be found in tropical smallholder systems, few studies provide empirical support for the integration of selective timber harvesting with non-timber forest product (NTFP) extraction. Brazil nut (*Bertholletia excelsa*, Lecythidaceae) is one of the world’s most economically-important NTFP species extracted almost entirely from natural forests across the Amazon Basin. An obligate out-crosser, Brazil nut flowers are pollinated by large-bodied bees, a process resulting in a hard round fruit that takes up to 14 months to mature. As many smallholders turn to the financial security provided by timber, Brazil nut fruits are increasingly being harvested in logged forests. We tested the influence of tree and stand-level covariates (distance to nearest cut stump and local logging intensity) on total nut production at the individual tree level in five recently logged Brazil nut concessions covering about 4000 ha of forest in Madre de Dios, Peru. Our field team accompanied Brazil nut harvesters during the traditional harvest period (January-April 2012 and January-April 2013) in order to collect data on fruit production. Three hundred and ninety-nine (approximately 80%) of the 499 trees included in this study were at least 100 m from the nearest cut stump, suggesting that concessionaires avoid logging near adult Brazil nut trees. Yet even for those trees on the edge of logging gaps, distance to nearest cut stump and local logging intensity did not have a statistically significant influence on Brazil nut production at the applied logging intensities (typically 1–2 timber trees removed per ha). In one concession where at least 4 trees ha^-1^ were removed, however, the logging intensity covariate resulted in a marginally significant (0.09) P value, highlighting a potential risk for a drop in nut production at higher intensities. While we do not suggest that logging activities should be completely avoided in Brazil nut rich forests, when a buffer zone cannot be observed, low logging intensities should be implemented. The sustainability of this integrated management system will ultimately depend on a complex series of socioeconomic and ecological interactions. Yet we submit that our study provides an important initial step in understanding the compatibility of timber harvesting with a high value NTFP, potentially allowing for diversification of forest use strategies in Amazonian Perù.

## Introduction

Timber often overshadows other components of the tropical forest use continuum, due to its high economic return (see [1–3]). Indeed, for several decades, the concept of sustainable forest management (SFM) was dominated by the idea of timber as a principal product [4–8], a trend possibly fueled by projected low net returns from non-timber forest products (NTFPs) in the 1990s (see [9–11]). Yet, many of the seminal papers on SFM focused on industrial logging, rather than logging by smallholders in forest communities, where forest valuation can be difficult to assess [12]. Exclusively focusing on timber (particularly in the case of smallholder systems) fails to capture other important forest products and ecosystem services [13]. Multiple-use forest management (see [14]) by contrast, recognizes the complex nature of diversified livelihood strategies and underscores the importance of looking beyond timber production as the only management objective [15–18].

Smallholder livelihood strategies related to wood extraction, farming and NTFP extraction have never been carried out in isolation [19–21]. Yet from a formal management standpoint, the implementation of multiple forestry objectives remains elusive on the ground, due to often acute tradeoffs among technical, socioeconomic, regulatory and normative aspects [22]. In particular, recent studies have highlighted both constraints and potential benefits for integrated NTFP and timber extraction. In some cases, NTFP harvests are a relatively safe diversification and risk management strategy in concert with timber removal (see [3, 23–27]. Especially as timber volumes dwindle in community and smallholder forests, diversification of management strategies at the landscape scale will be critical.

Among the thousands of NTFP species that exist in the Western Amazon, *Bertholletia excelsa* (Humb. and Bonpl., Lecythidaceae), or Brazil nut, is currently one of the most economically valuable [28–29], contributing substantially to the annual gross domestic products (GDPs) of Peru, Brazil, and Bolivia [30]. Indeed, it is one of the few tropical tree species to have had a lasting impact on forest legislation across the region—it is illegal to fell Brazil nut trees in Brazil, Bolivia, and Peru [18, 31–32]. Often touted as a keystone species for integrating sustainable development and conservation, Brazil nut has the distinction of being the only globally-traded seed crop collected by forest-based extractivists [28, 31]. Many smallholders in Madre de Dios (Perú), Acre (Brazil), and Pando (Bolivia), the so-called “MAP” region [33], have historically depended on Brazil nut harvests and other NTFPs (e.g., rubber; *Hevea brasiliensis*, Euphorbiaceae) to support their families [34–38].

Although recent papers have considered the integration of Brazil nut and selective timber harvesting [18, 24, 26, 38–40], no research to-date has assessed *B*. *excelsa* fruit production in selectively logged forests in order to inform sustainable use of these coexisting forest products. Already, selective logging activities are known to alter inter-tree pollen flow, damage the soil and the residual forest, as well as reduce soil moisture [6, 41–44]. Several of these examples from the literature originate from forests where logging intensities are typically high (≥ 15 m^3^ ha^-1^), yet at least physical damage to adult trees (including Brazil nut; see [45]) has been observed in smallholder systems [45] and industrial concessions [24] at low logging intensities (5–10 m^3^ ha^-1^). Alteration of tree fruiting patterns in selectively logged forests has also been noted, yet almost solely within the context of conspecifics being physically removed [46–49]. In contrast, the impact of timber species removal on (tree-based) NTFP taxa fecundity has rarely been studied (reviewed in [50]). Selective logging can stimulate the production of tree fruit in neighboring individuals, possibly due to enhanced light conditions after tree harvesting (e.g., [48, 51], but see [52]). Conversely, tree fruit production could decrease as effective population sizes dwindle concurrently with disruptions in pollinator behavior (albeit depending on pollinator species and spatial pattern of logging gaps), particularly in outcrossing species (e.g., [53]). In this paper we ask the following question: To what extent is Brazil nut production at the individual level affected in a logged forest landscape? We frame this question based on covariates related to the logging activities (i.e., distance of productive Brazil nut trees to both nearest cut stump and local logging intensity) while controlling for tree-based variables known to influence Brazil nut fruit production.

## Materials and Methods

### Study site

**Fig 1:**
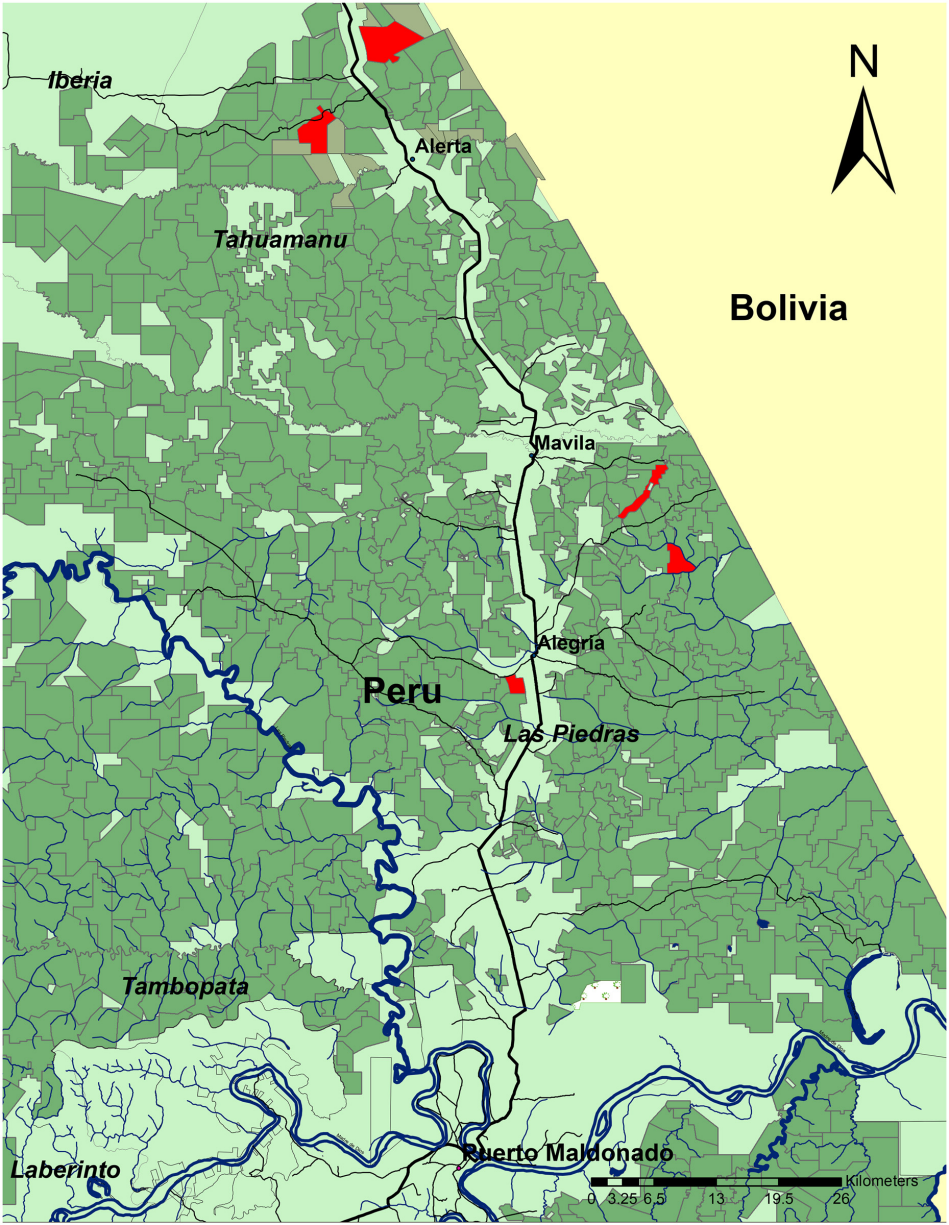
Location of study area (Madre de Dios, Peru). Dark green outline: Brazil nut concessions; Red outline: Participating Brazil nut concessions.

**Fig 2:**
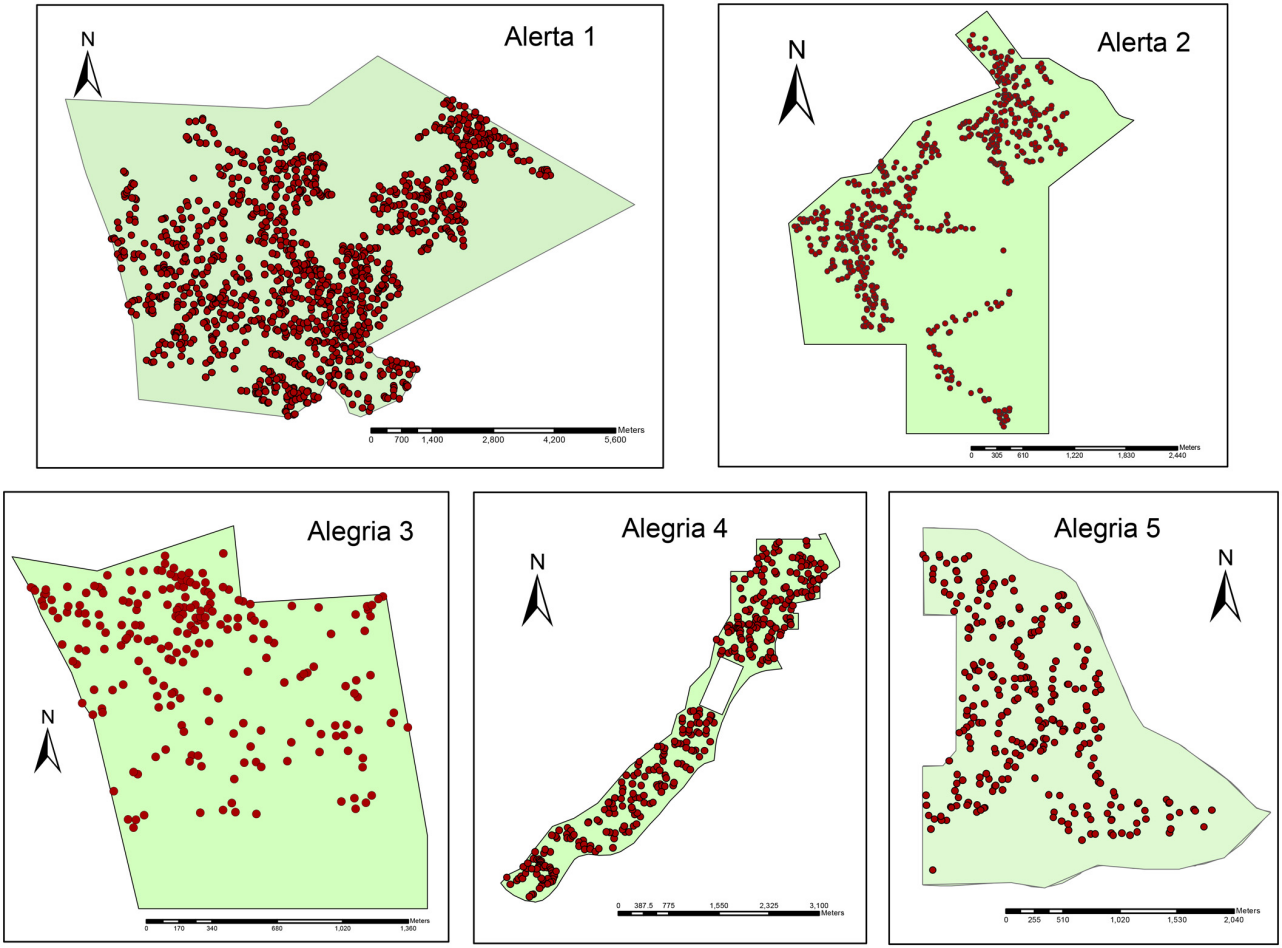
Spatial distribution of Brazil nut trees ≥ 40 cm DBH in five Brazil nut concessions (290–1750 ha), Madre de Dios, Peru. The 499 individual trees included in the analysis were randomly selected from these populations. Brown circles: Brazil nut adult trees.

The study was conducted in five Brazil nut concessions (ranging from 290 to 1750 ha; Figs 1 and 2) in southeastern Peru, Tahuamanu and Las Piedras Provinces, Department of Madre de Dios (11°30”30” -12°10’0” S and 69°56’0”-69°21’0” W), from January 2013 to April 2014. Permission to work in the concessions was given by the five Brazil nut concessionaires (*castañeros*) who hold title to the 40-year state-owned concessions (see [54]). Mean annual rainfall ranges from 2500 to 3500 mm, with a pronounced rainy season from December to March [39, 55]. Mean annual temperature is 24°C, but it fluctuates within an extreme range for the neotropics (10–38°C) [55]. The landscape is defined by acidic, well-drained soils of moderate to low fertility and gently undulating to flat topography. Vegetation along the Interoceánica Sur Highway (IOS; connecting the Peruvian coastal ports with Brazil and Bolivia) is characterized by seasonally moist *terra firme* lowland forest, often dominated by arborescent bamboo (*Guadua sarcocarpa* Londoño and P.M. Peterson and *G*. *weberbaueri* Pilg., Poaceae; [56–58]). Forest concessionaires associated with this study (see below) identified seven vegetation types located in their particular concessions: palm forest (*palmichal*); bamboo forest (*pacal*), upland forest (*bosque colinoso*), closed canopy forest (*bosque alto*), secondary forest (*purma*), moriche palm (*Mauritia flexuosa*, Arecaceae) forest (*aguajal*), and abandoned pasture (*potrero*).

Approximately 30% (~ 2.6 million ha) of Madre de Dios is characterized by Brazil nut-rich forests [39, 59], with an average reported density of 1.3–1.5 adult trees ha^-1^ [32]. Harvesting entails collecting fallen fruits, removing the outer shell, then drying and shelling the seed (not a true nut, as the common name would suggest; [54]). Brazil nut concessions (totaling about 1000 concessions and encompassing about 995,590 ha) were established in 2000 by the Peruvian Forestry and Wildlife Law no. 27308 as a way of formalizing traditional usufruct rights [32, 39, 40, 54]. The size of a Brazil nut concession in Madre de Dios ranges from 39–3900 ha (average: 850 ha). These management units are controlled by individuals or families who reside in the concession only during the Brazil nut harvest season, from January to March. Several researchers have noted low deforestation rates both around and within these Brazil nut concessions, highlighting their conservation value [33, 60].

Like Brazil nut harvesting, timber has also been a major source of income in the region for decades [32, 61– 62]. Presently, at least 80% of the timber in Madre de Dios is illegally harvested [63–64]. In recognition of the resource extraction activities that were already taking place on an informal basis, the government decreed in 2004 that timber could be legally harvested from Brazil nut concessions [32]. In order to log in their concessions, concessionaires must register with the Regional Government of Madre de Dios, to whom they must submit a separate management plan [(Plan Complementario para el Aprovechamiento de Madera (PCM)]. Reported timber harvesting intensities in the concessions have remained low (1–2 trees ha-^1^; [32, 64]) since the implementation of the 2004 decree. Nonetheless, more commercial timber volume is leaving Brazil nut concessions in comparison to designated industrial timber concessions, likely due to less stringent fiscal and bureaucratic requirements in the former. In 2010, for example, official records showed a total of 9630 m^3^ of timber was harvested from Brazil nut concessions in Madre de Dios, versus 3223 m^3^ from neighboring timber concessions [32, 65]. Brazil nut concessionaires typically harvest timber in an opportunistic fashion with multiple entries into the same harvest zone. Some of the more commonly exploited species include *tornillo* (*Cedrelinga catenaeformis*, Fabaceae), *sapote* (*Matisia* sp., Malvaceae), and *lupuna* (*Ceiba pentandra*, Malvaceae) [32]. Due to historical heavy exploitation, many internationally valuable hardwood species[e.g., *tahuari* (Brazilian walnut, *Tabebuia* spp., Bignoniaceae) and *shihuahuaco* (Brazilian teak, *Dipteryx* spp., Fabaceae)] are extremely rare in Brazil nut concessions within close proximity to the IOS.

History of timber extraction varies amongst the five concessions, with at least one concessionaire stating that she only started cutting timber fairly recently (in 2006) and now harvests on a yearly basis. Another concessionaire started harvesting in 2007, did not harvest again until 2010, and now harvests on a yearly basis. All concessionaires claim to avoid Brazil nut groves when engaging in logging activities, although in many cases it is often a *tercero*, or outside logging crew, that is harvesting the timber, not the concessionaire. When concessionaires were asked if they thought that the *tala*, or timber harvesting, had an impact on the Brazil nut trees, all concessionaires mentioned the impacts in terms of benefits and disadvantages. Benefits could include a quick release of nutrients for the Brazil nut trees, and while one respondent mentioned that production levels might initially decrease as a result of the timber extraction, production usually recovers within a short period of time. Three informants mentioned that wind damage often occurs as a result of removing large-canopy trees from the forest.

### Species description

*Bertholletia excelsa* is a long-lived canopy emergent, with some individuals estimated to be 500–1500 years old [66–69], reaching 60 m in height and 16 m in circumference [70–71] and providing key resources for several animal species (see [72–74]). It is distributed across the Amazon Basin most often in groves of 500–100 individuals in *terra firme* forests [75–77]. Evidence suggests that the species’ basin-wide distribution is the product of deliberate anthropogenic intervention [77], although short-distance nut dispersal by caviomorph rodents also plays a role [78]. Most estimates of the species’ density [diameter at breast height (DBH) ≥ 10 cm] range from 1–3 stems ha^-1^ (e.g., [31, 76, 79–80]), although some estimates are as high as 23 individuals ha^-1^ [71].

*B*. *excelsa* is an allogamous, self-incompatible species with reportedly high levels of genetic diversity and inter-tree pollen flow at the population level [81]. Its flowers are pollinated by medium to large sized bees of different genera: *Bombus* [82], *Euglossa* [83], *Eulaema* [84], and *Xylocopa*; the latter two being the most frequent flower visitors [84–86]. The large-bodied (10–16 cm; [83]) fruit requires 14–15 months to mature [85] and contains 10–25 large seeds (2x5 cm [76]). Fruit production varies with DBH, degree of crown illumination, crown form and crown diameter [87– 91], presence of vines [31, 92], and it also shows high interannual variability at the individual level [87, 90]. Seeds take a relatively long time (12–18 months) to germinate, likely due to the impenetrable, woody nature of the pyxidium [77].

### Sampling design

Brazil nut concessionaires were asked to participate with the project during meetings conducted in October 2012 with concessionaire associations in the settlements of Alegría and Alerta (Fig 1). Although a list of 12 association members was generated during these meetings, the final number of interested concessionaires was reduced to five. This decision was primarily based on concession accessibility, number of available field technicians (at least two to every concession), and compatible schedules between the field team and Brazil nut harvesters, particularly during the peak of the Brazil nut harvest (January-April). Prior to the selection of the five concessions, we had no information about the timber harvest intensities being applied across these sites.

We did not employ the “logged/unlogged” design applied in most selective logging studies (e.g., [6, 48]) for various reasons. First, few Brazil nut concessions close to the IOS are considered “unlogged” and thus suitable as control sites. Furthermore, the small size and narrow shape of some of the study concessions meant very few areas were independent of the influence of selective timber harvesting impacts (Fig 1). Our previous observations across the study area also showed that selective timber harvesting in Brazil nut concessions does not follow a polycyclic model (cf. [32]), in which a series of unlogged compartments remain undisturbed over the course successive cuts. This trend was further confirmed when we examined a random sample of officially-approved timber harvesting plans in these concessions. It was evident, however, that the spatial extent of selective logging was highly heterogeneous across all concessions.

### Spatial attributes

In order to measure the impact of selective logging on a particular biophysical response variable (e.g., tree damage, tree growth rates), distance to origin of the logging disturbance is often employed as an independent factor (see [45, 93]). As such, to characterize the logging mosaic in each concession and assess the potential impact of selective logging on fruit production for a given tree, distance to nearest logging gap (cut stump), and nearest local logging intensity (trees removed ha^-1^) were measured. For each of the five concessions, we mapped all existing logging gaps (≤ 5 years old) in the presence of the concessionaire, who confirmed the history of each gap. Although we also mapped all skid trails onsite, given that change in canopy opening (and thus, light availability) was more pronounced in the logging gaps, we determined that distance to cut stump was the most appropriate covariate for inclusion in the analysis. All distance measurements were calculated using geo-referenced locations in ArcMap 10.1 (UTM Zone 19S; distance to nearest conspecific neighbor ≥ 40 cm DBH and distance to nearest cut stump). All logging gaps (≤ 5 years old) were also geo-referenced in ArcMap 10.1. Local logging intensity was calculated in ArcMap 10.1 by determining the number of cut stumps within a 56.4 m (equivalent to 1 ha) radius around each individual cut stump. Distance to nearest conspecific reproductive neighbor was also measured to account for potential variation in fruit production, given that the species relies on pollen out-crossing for reproduction.

### Fruit and nut production

The study comprised a random selection of 499 Brazil nut trees (≥ 40 cm DBH, the minimum size visited by Brazil nut harvesters and minimum DBH for reproduction in closed-canopy forest; [90]) across the five concessions (comprising about 4000 ha of Brazil nut-rich forest; Fig 2). The sample was obtained by generating a randomized sequence of integers, using the identification number of each individual tree visited in all five concessions with the harvesters (total of 1443 trees). The total number of trees visited by our field team and Brazil nut harvesters was not a 100% coverage of each concession, although we did attempt to include at least 100 trees from each concession in the analysis, including those individuals that failed to produce fruit for a particular year. Most of the trees were within close proximity of conspecific neighbors (see Fig 2), as the Brazil nut harvesters with whom we were working often abandoned the harvest of more isolated trees, especially in the larger concessions. Each individual tree was considered as a replicate for the analysis and logging impacts on fruit production were assessed by measuring the distance from a productive Brazil nut tree in our sample to the nearest logging gap and its associated logging intensity (number of stumps ha^-1^).

**Fig 3:**
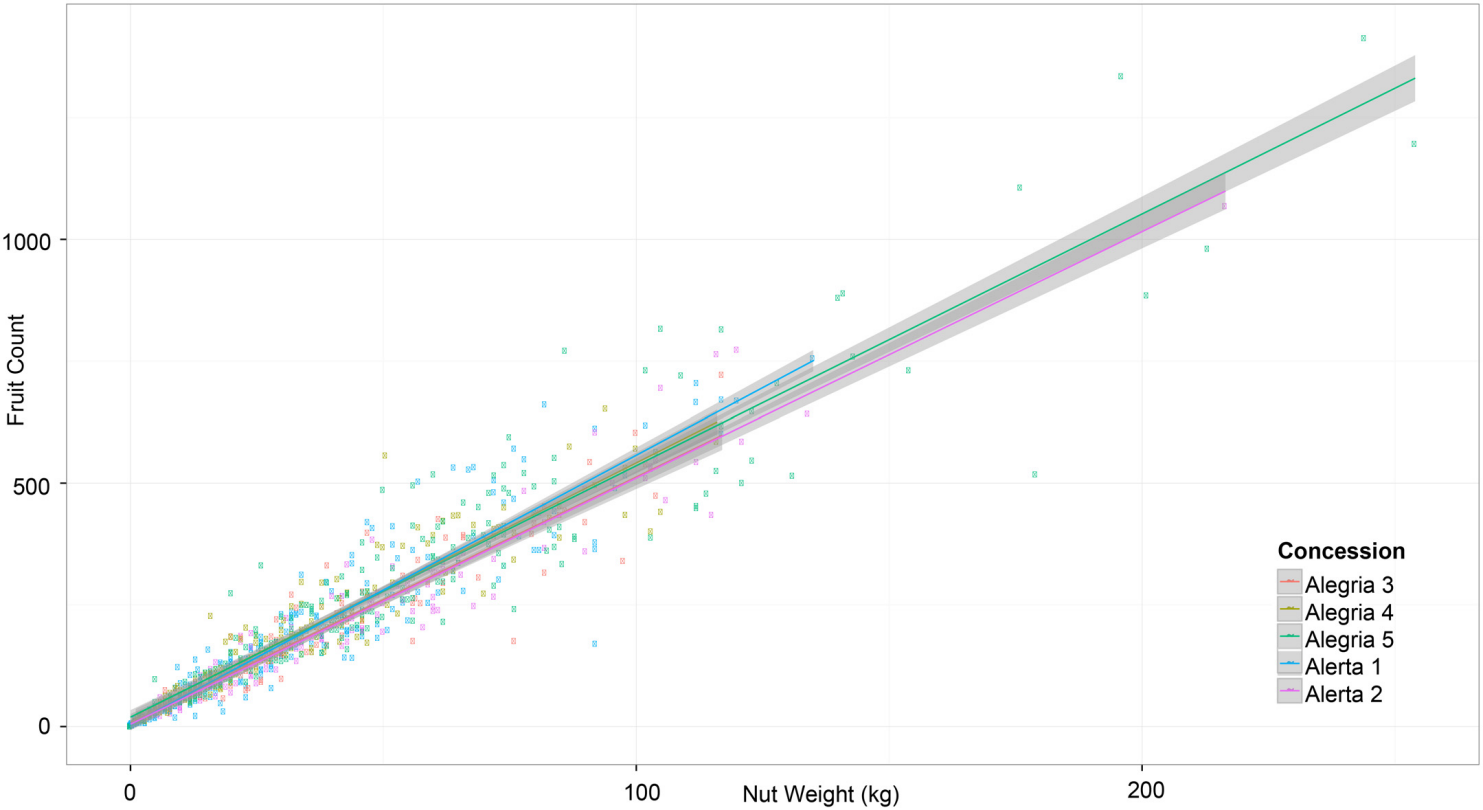
Scatter plot of relationship between total fruit count and total nut weight (kg), with best fit line and 95% CI. Circles represent 499 Brazil nut trees (≥40 cm DBH), color coded by site (concession).

Fruit and nut production of the selected trees was monitored in all five concessions during the 2013 and 2014 harvest seasons (January-April). Individual Brazil nut trees were located and geo-referenced with a Garmin GPSMap 62 handheld unit. They were subsequently measured for DBH at 1.3 m above the ground, using a fabric diameter tape. Our field team followed Brazil nut harvesters as they collected fruits, dividing them accordingly on the ground under the appropriate “mother” tree. Fruits were then counted for each tree, their outer shells cracked using a machete, and the nuts produced per tree were weighed with a spring balance before being mixed with nuts from other trees in large sacks, or *barricas*. One *barrica*, the traditional method for transporting the product to local middlemen, is the equivalent of approximately 70–80 kg. As nut weight is an important local measurement of Brazil nut productivity, we chose to focus on total nut weight per tree as our dependent variable, similar to other Brazil nut studies (see [31]). The size of fruit and number of seeds per fruit also varied considerably among trees, leading us to believe that nut weight was a more consistent measurement of local Brazil nut tree productivity than fruit count (see Fig 3). In a completely controlled experiment, the ideal method for nut weight measurement would have been to subtract oven-dried weight from the fresh weight measurements taken in the field. Since our field team was obliged to follow local nut harvester protocol and process the nuts immediately before they were taken to buyers for distribution, we took measurements directly in the field. While this method may have resulted in a slight overestimation in weight measurements due to elevated moisture levels, since nuts in all five concessions were harvested contemporaneously, the error would have been consistent across the sites. We also attempted to reduce sampling error by passing by each tree at least twice to search for fruit. As noted by Zuidema and Boot [90] and Staudhammer et al. [94], the number of fruits collected from the ground does not reflect a 100% harvest, due to predation and incomplete harvests. Indeed, remaining fruits in the crown of a tree will fall as late as May or June, which is why the field team remained in the sites later than the nut harvesters, verifying that there were no additional fruits on the ground.

### Tree attributes

Recognizing that several key tree-based independent variables are already known to influence fruit production, we measured tree crown diameter, crown position, crown form, trunk form, assessment of damage, and liana load (see [31, 87, 94]). Tree crown diameter was determined by measuring the longest spread and the longest cross-spread with a 50 m tape underneath the crown of the tree. Average crown spread of the Brazil nut tree was then calculated for each tree by adding the value of the longest spread with the value of the longest cross-spread, divided by 2. Crown position was determined using a modified Dawkins illumination index cited by Synnott [95] and Wadt et al. [31]: (1) dominant (full overhead and side light); (2) co-dominant (full overhead light); (3) intermediate (some overhead and lateral light); (4) < 10% lateral light; (5) suppressed (no direct light). Crown form was also adapted from the Dawkins method: (1) complete circle; (2) irregular circle; (3) half-crown; (4) less than half crown; and (5) few branches. Description of trunk form was established using methods developed by the field team: (1) trunk well-formed, straight; (2) trunk leaning; (3) trunk damaged. Assessment of damage was evaluated using binary categories: (0) no damage; and (2) damage. When it was possible to determine that the damage was directly due to logging activity, this observation was noted, but since we were unable to collect “before” data, we could not determine damage status (from logging/other causes) with 100% certainty. As such, we did not make the distinction in the analysis. Similarly, we used a binary categorization for liana and nail presence: (0) no liana/nail; and (2) liana/nail. It is common lore amongst Brazil nut harvesters in the region that the presence of a nail will cause an individual tree’s productivity to drop. Nails are often used to secure tree tags to the trunk when conducting inventories, but this practice is becoming less common as a result of this long-held belief.

### Data analysis

A repeated measures general linear mixed effects model was used to evaluate effects of the fixed independent factors (distance to nearest cut stump, local logging intensity, distance to nearest conspecific neighbor, DBH, crown diameter, crown position, crown form, trunk form, assessment of damage, presence of liana, presence of nail) on total nut weight per tree in 2013 and 2014. Based on results from a long-term study in Acre, Brazil [31], as well as patterns determined from our own data set, DBH^2^ was included in the model to account for the quadratic relationship between DBH and fruit production. Site was included in the models as a random factor. Analyses were conducted using the lme4 package of the R 3.10 software platform [96]. Data were log 10 transformed to conform to assumptions of normality and homoscedasticity. Results were considered statistically significant at P ≤ 0.05.

## Results

### Stand-level and tree attributes

The total number of Brazil nut trees (≥ 40 cm DBH) in all five concessions was determined to be 1741 trees (Alerta 1), 1051 (Alerta 2), 291 (Alegría 3), 547 (Alegría 4), and 576 (Alegría 5). Local stem density for the same size class was calculated as 0.68 trees ha^-1^ (Alerta 1), 0.54 trees ha^-1^ (Alerta 2), 0.75 trees ha^-1^ (Alegría 3), 0.57 trees ha^-1^ (Alegría 4), and 0.58 trees ha^-1^ (Alegría 5). Mean distance between conspecific reproductive trees was calculated for the five concessions: 56.9 m (Alerta 1), 70.7 m (Alerta 2), 58.1 m (Alegría 3), 60.5 m (Alegría 4), and 81.1 m (Alegría 5).

**Fig 4:**
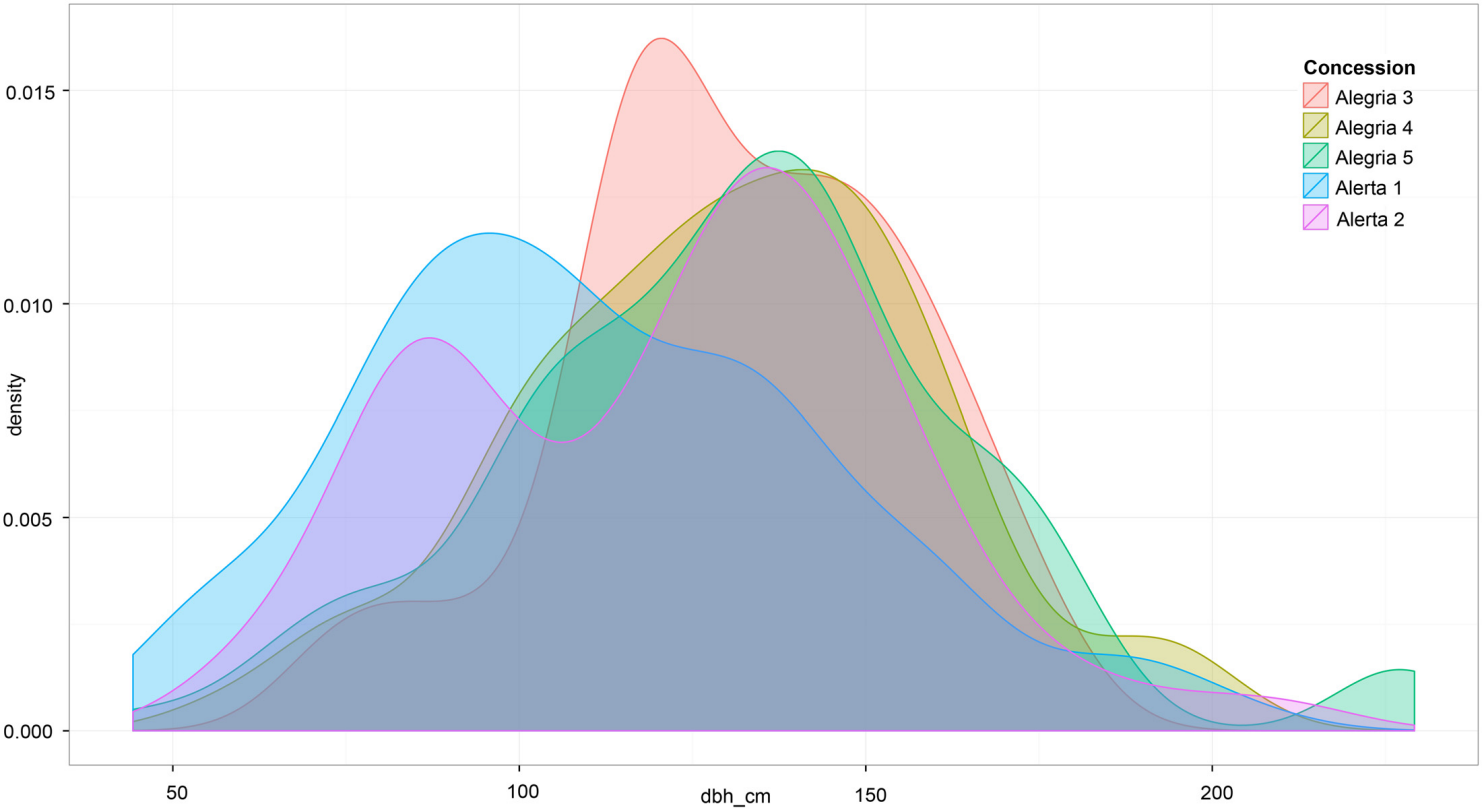
Density plot, or the estimation of the probability density function (Y) of diameter (DBH, X) of 499 Brazil nut trees (≥40 cm DBH).

**Fig 5:**
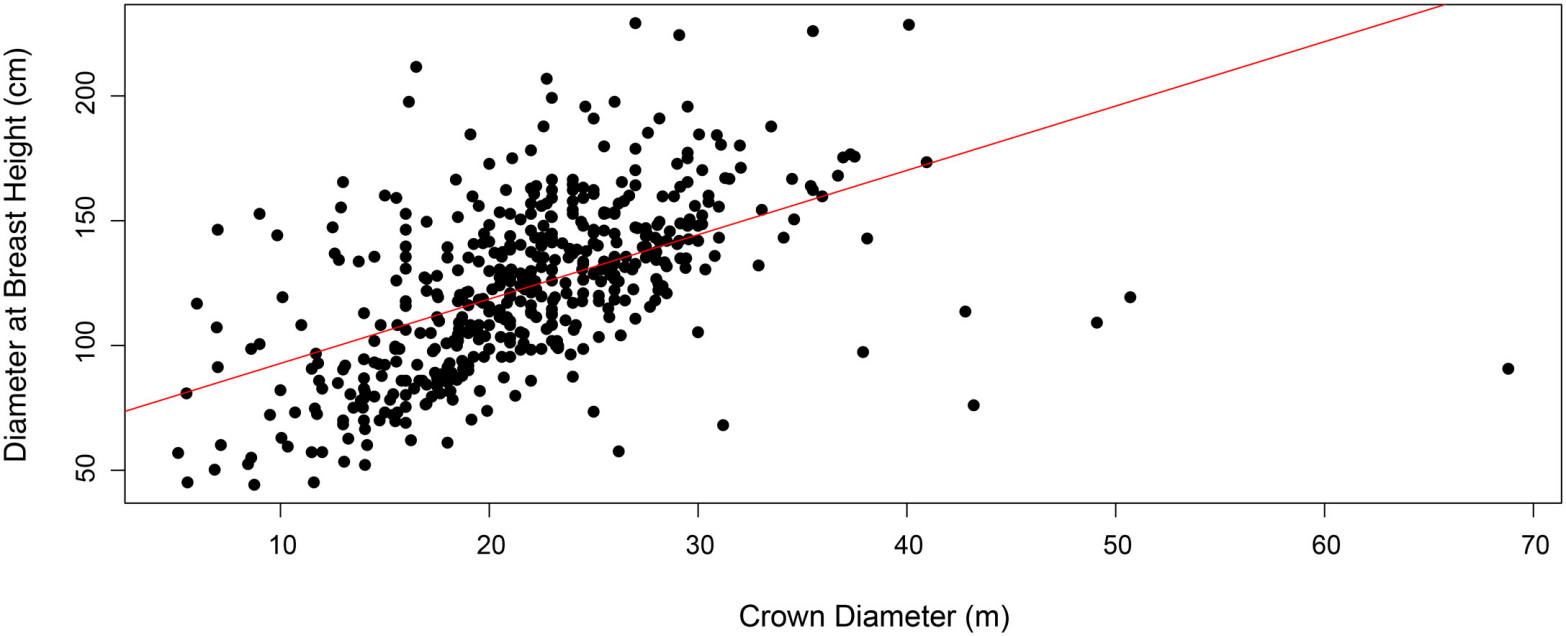
Scatter plot of relationship between diameter at breast height (cm) and crown diameter (m), with best fit line and 95% CI.

Average diameter (≥ 40 cm DBH) found in each of the five concessions (using data from our sample of 499 Brazil nut trees) was 111.7 cm (Alerta 1), 122 cm (Alerta 2), 131 cm (Alegría 3), 129 cm (Alegría 4), and 132 cm (Alegría 5). In all 5 concessions, DBH ranged from 44.2 to 229.2 cm DBH (Fig 4). The relationship between crown diameter and DBH was significant, but not tightly correlated; that is, the crown size was not always a consistent predictor of DBH (Fig 5). Of the 499 trees included in this study, only 1% was ≤ 50 cm DBH and just over 20% had a diameter ≥ 150 cm DBH. We found that a relatively low percentage of the Brazil nut individuals were characterized by a “perfect” crown form—34%. Yet, of the 499 trees, there were very few trees with poor crown quality (half a circle or less = 26). Slightly less than half (43%) of the trees were determined to be of the dominant crown position category; only three individuals were described as suppressed.

### The logging landscape

Mean distance from a given Brazil nut individual included in the analysis to the nearest logging gap was substantial: 364 m. Logging intensity varied strongly between the sites, with some concessions centralizing their activities in selected areas, as opposed to an even distribution of logging activity across landholdings. All concessions engaged in multiple tree ha^-1^ extraction. However, we found this more intensive logging strategy to be a rare event in all five concessions, with most extraction limited to 1–2 trees ha^-1^. Recently felled trees, logged ≤ 5 years prior to the study (2013), numbered 40 stumps in Alerta 1, 85 stumps in Alerta 2, 14 stumps in Alegría 3, 39 stumps in Alegría 4, and 37 stumps in Alegría 5.

### Fruit and nut production

**Fig 6:**
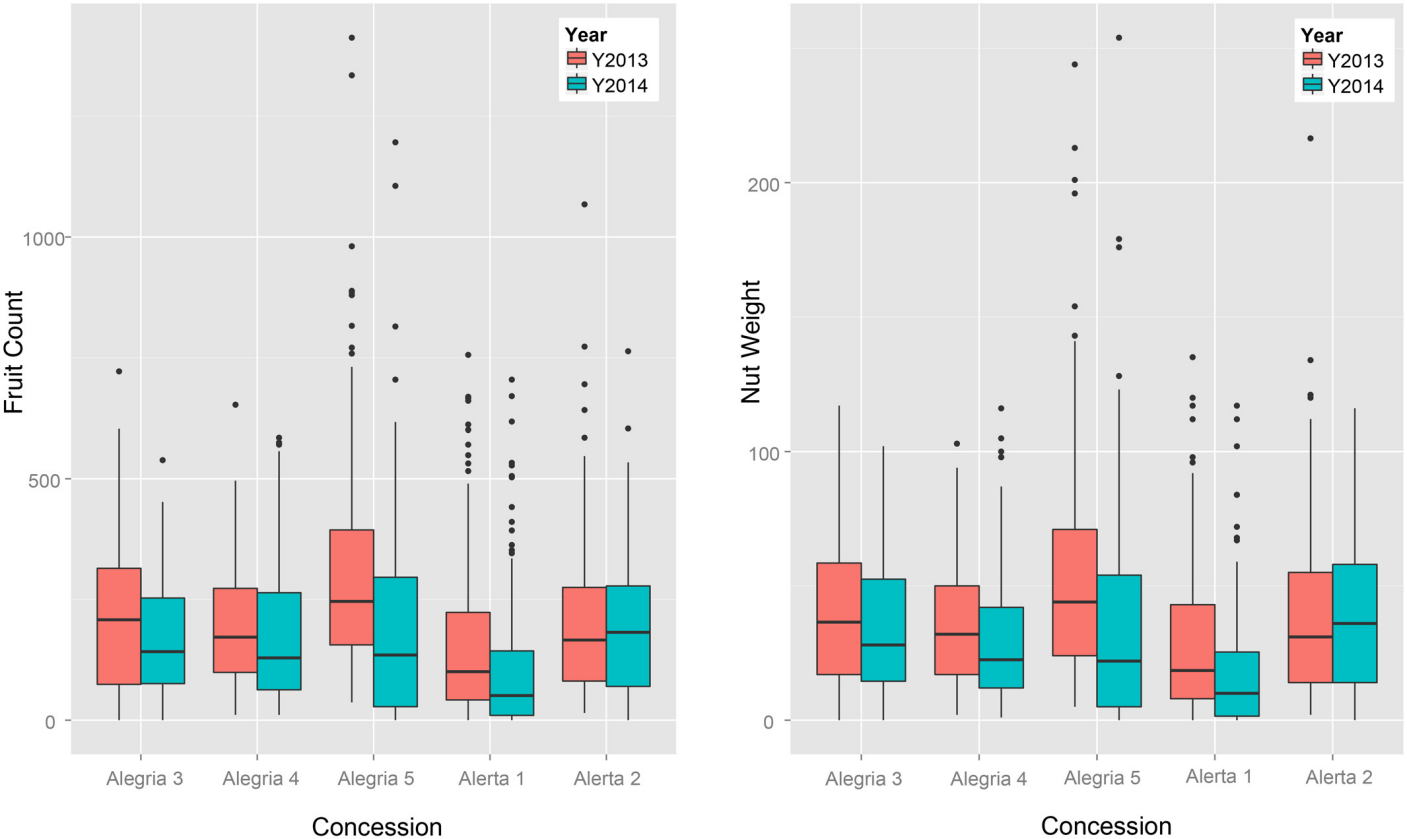
Distribution of total fruit count and nut weight (kg) per tree for 499 Brazil nut trees (≥40 cm DBH).

Median fruit production per tree in 2013 was calculated as 159 fruits (Alerta 1), 216 fruits (Alerta 2), 218 fruits (Alegría 3), 197 fruits (Alegría 4), and 316 fruits (Alegría 5) (Fig 6). Overall mean fruit production per tree calculated from all five concessions was 218.5 fruits per tree. For those trees located more than 100 m away from the nearest conspecific (≥ 40 cm DBH), the mean fruit count per tree was 232. 8. In 2014, the average count was the following: 108 fruits (Alerta 1), 201 fruits (Alerta 2), 175 fruits (Alegría 3), 179 fruits (Alegría 4), and 200 fruits (Alegría 5), with the overall mean value per tree dropping to 163.3 in 2014 (Fig 6). Remote trees (≥ 100 m from nearest conspecific) produced, on average, 165.6 fruits per tree. Only eight trees in the study produced no fruits in 2013, while 48 were unproductive in 2014 (including four of the unproductive trees from 2013). Of those unproductive trees in 2014, 46% were from the Alegría 5 concession, and all had produced fruit the previous year. Median nut weight per tree across the five concessions in 2013 was 29.03 kg (Alerta 1), 41.8 kg (Alerta 2), 40.4 kg (Alegría 3), 35.05 kg (Alegría 4), and 55 kg (Alegría 5) (Fig 6). Overall nut weight per tree across the five concessions in 2013 was determined to be 39.4 kg, while those trees labeled as remote produced, on average, 28.5 kg per tree. In 2014, the mean nut weight per tree for the five concessions was calculated as 18.3 kg (Alerta 1), 38.8 (Alerta 2), 34 kg (Alegría 3), 30.5 kg (Alegría 4), and 37.5 kg (Alegría 5), with a median value of 29.6 kg per tree for all five concessions (Fig 6). The mean nut weight per tree for remote trees in 2014 was 28.5 kg. Fruits were sometimes smaller than 10 cm in circumference, compared to the more standard 10–16 cm, causing a considerable amount of variability when comparing total nut weight with total fruit count (Fig 3).

**Fig 7:**
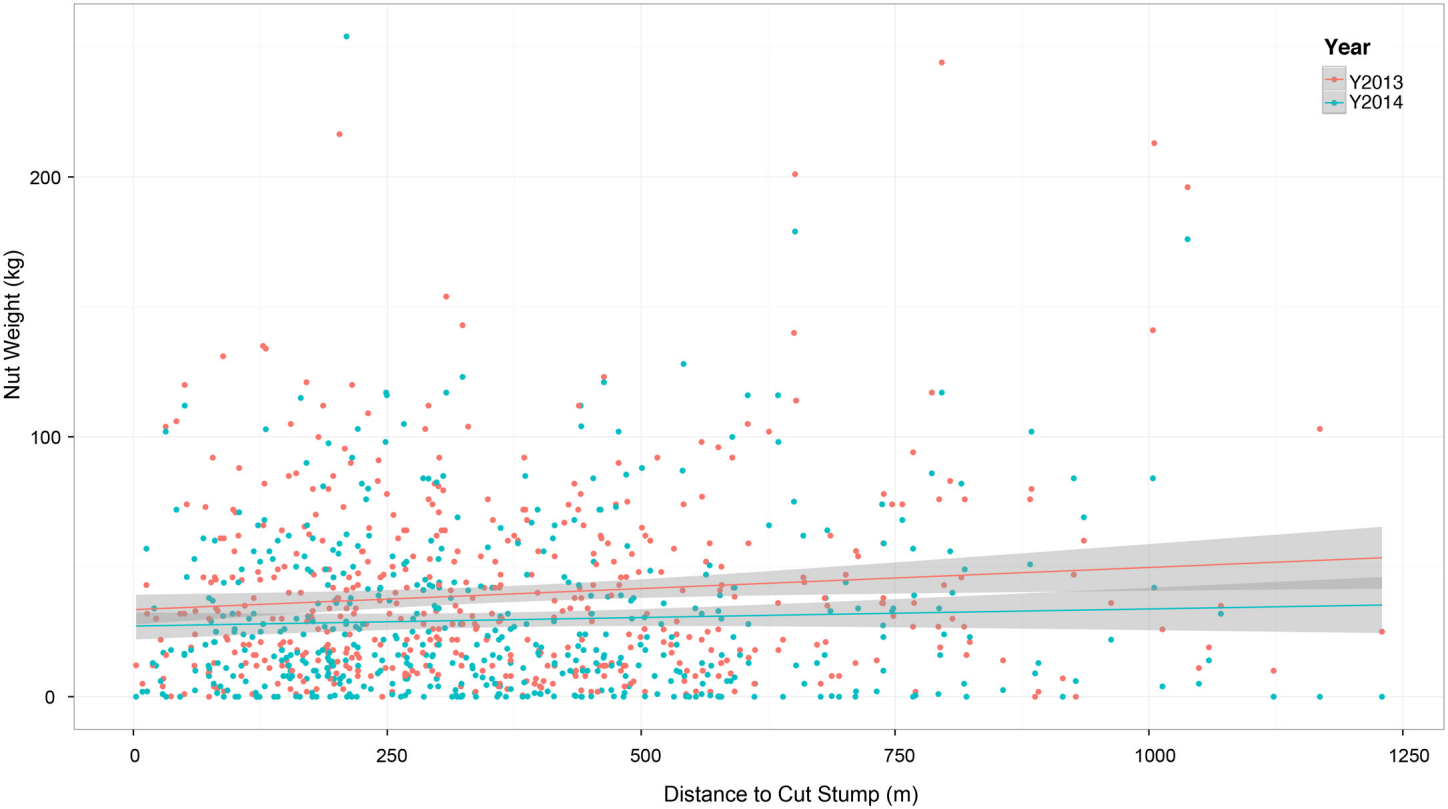
Scatter plot of relationship between distance (m) to nearest logging gap (cut stump) and total nut weight (kg), with best fit line and 95% CI.

Significant production variation was detected by year (P≤ 0.0001), a finding that remains consistent with other studies from the region and information gathered from local Brazil nut concessionaires. Additionally, the following independent variables were identified as strong influences on total nut weight per tree: year, crown diameter, crown form, crown position, and presence of liana (Table 1). Our logging-related covariates did not prove to be significant sources of variation for total nut weight per tree [P = 0.54 (distance to nearest cut stump), P = 0.09 (local logging intensity); Table 1, Fig 7]. However, we noticed that these effects were more pronounced in one particular concession, Alegría 4. In contrast to the other concessions, Alegría 4 demonstrated several instances of high logging intensities (3–4 trees ha^-1^). When the data from this concession were analyzed independently of the other concessions we discovered that distance to logging gap and local logging intensity had a negative influence on an individual tree’s total nut weight. We could not duplicate these results in other concessions, given the low logging intensities.

**Table 1:** Linear repeated measures mixed effects model analysis with total nut weight per tree as dependent variable.

	Estimate	Standard error	Z value	Pr(>|z|)
(Intercept)	3.26	1.04	3.14	≤0.01
DBH	-0.00	0.01	-0.40	0.70
Log Dist. Logging Gap	-0.10	0.17	-0.61	0.54
Year	-0.54	0.07	-7.70	≤0.0001**
Intensity 2 trees ha^-1^	-0.01	0.10	-0.07	0.94
Intensity 3 trees ha^-1^	-0.11	0.29	-0.37	0.72
Intensity 4 trees ha^-1^	-0.69	0.41	-1.69	0.09
Crown diameter	0.05	0.01	6.57	≤0.0001**
Crown form (few branches)	-1.28	0.57	-2.25	0.03*
Crown form (full circle)	-0.73	0.41	-1.75	0.08
Crown form (half circle)	-0.70	0.45	-1.55	0.13
Crown form (irregular circle)	-0.83	0.42	-2.01	0.03*
Crown position (dominant)	0.04	0.12	0.36	0.72
Crown position (intermediate)	-1.00	0.41	-2.42	0.05*
Crown position (suppressed)	0.04	0.48	0.08	0.94
Distance nearest neighbor	-0.00	0.00	-1.17	0.24
Presence liana	-0.40	0.15	-2.71	≤0.01**
Presence nail	0.04	0.08	0.43	0.69
Presence damage	0.29	0.15	1.90	0.06
DBH*Log Dist. Gap	0.00	0.00	0.94	0.35

## Discussion

### Variation in total nut weight in relation to logging

Our study specifically targeted the influence of proximity to logging gaps on total nut weight of *Bertholletia* individuals, taking into account both tree and stand level attributes. Across two consecutive fruiting seasons, we saw no significant change in overall per tree nut weight measurements with reference to distance to closest logging gap or logging intensity when data from all five concessions were analyzed together. This result is not completely surprising, since 399 of the (randomly-selected) 499 Brazil nut trees included in this study were located more than 100 m away from the closest logging gap, suggesting that concessionaires (corroborated during the interviews) are successfully avoiding Brazil nut trees when carrying out logging operations. A very large proportion of Brazil nut trees in our sample had well-illuminated crowns (a requisite for commercially-viable reproduction in natural forest stands for this species; [90]). This trend indicates that fruit production in these individuals might be insensitive to logging-mediated canopy disturbances, especially when low intensity logging activities are conducted at least 50 m from the stem in question. And even though we are uncertain as to the source of damage (given that we have no pre-logging data before 2013), damage levels were not significant to the point of being an explanatory factor in fruit production.

Although low logging intensities (1–2 trees ha^-1^) are generally the norm in Madre de Dios [32, 65], we did see evidence of multiple-tree logging gaps in the case of Alegría 4. Indeed, when a logging crew reaches a particular part of the forest where several timber trees are located, it is more economical to take all of these stems at once, rather than just one or two of them. To recuperate the cost of the trees left behind, the logging crew needs to look for trees of similar value in other parts of the forest, using up worker hours as well as fuel. This type of situation is typically characteristic of unplanned operations (see [6, 97]). While not statistically significant, a P-value of 0.09 (see Table 1) for the logging intensity variable in this study might be a call for forest managers to proceed with caution when harvesting at high intensities in Madre de Dios. Indeed, when data from Alegría 4 were analyzed on their own, local logging intensity was strongly linked to lower production levels. For example, when comparing the difference in percent yield between Brazil nut trees associated with a local logging intensity of 1 tree ha^-1^ and stems associated with an intensity of 4 trees ha^-1^, there was a 68% difference in the mean nut weight values. Other studies have pointed to reduced soil moisture levels in large gaps [98] and declines in inter-tree pollinator movement [52] due to large gap openings. Thus, it is possible that increased harvest intensities could induce similar biophysical changes in these forests, altering the process of fruit production in *Bertholletia* (see below).

### Other explaining factors

Given *Bertholletia*’s self-incompatible reproductive system, we initially expected fruit production to be influenced by distance to nearest conspecific neighbor, but this variable turned out to be a poor predictor of total nut weight per tree (P = 0.24). Ghazoul et al. [53]’s study of *Shorea siamensis* (Dipterocarpaceae), another insect pollinated, self-incompatible species, demonstrated that fruit production declined once reproductive conspecifics become isolated due to selective logging. In contrast, Brazil nut trees are protected from felling, and thus there is no risk of reproductive individuals becoming isolated from their conspecific neighbors. Since the average distance between reproductive conspecifics of *Bertholletia* across all five concessions did not exceed 90 m, bee-mediated pollen flow is probably not constrained at our study sites. Yet (even though Brazil nut's pollinators can fly over long distances; see [99]) a multiple tree logging gap located within close proximity of a reproductive Brazil nut stem might alter inter-tree, flower visitation rates. More complex analyses may be needed to better distinguish the effect of tree population density (i.e., distance to groups of conspecific neighbors, rather than distance to single neighbors) as well as extent of canopy disturbance, on fruit production. It is quite possible that fruit set will be higher for those trees located in a cluster of neighbors, rather than those with a single close neighbor.

It is important to note that fruit production is highly variable for many tropical forest tree species (including Brazil nut; [90, 100]), and defining a “typical” year of production is constrained by lack of long-term production data. Accordingly, we saw a significant difference (P ≤0.0001) in average total nut production per tree between 2013 and 2014. We also observed an effect of site (concession) on total fruit production per tree. The Alerta 1 concession was consistently the poorest fruit production in both 2013 and 2014 (Fig 5). One explanation is that this concession is dominated by *Guadua* spp. (*pers*. *obs*.), an arborescent bamboo that is a powerful competitor with even the largest canopy emergents [56–57]. These results confirm local concessionaires' observations on geographic and temporal variability on tree fecundity, as well as Brazil nut collectors across the Amazon Basin [29, 101], and other researchers [32, 87, 92].

**Fig 8:**
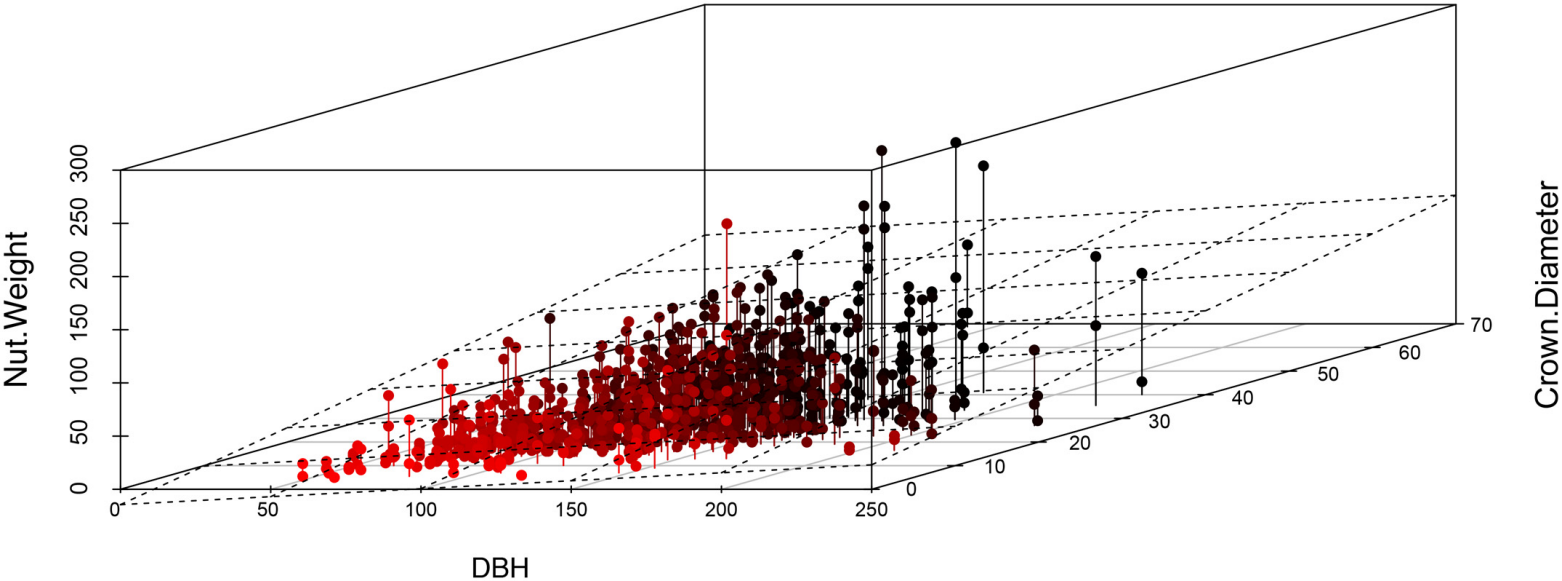
Observed nut weight (kg) data across a range of diameter at breast height (cm) and crown diameter (m) values.

Concurring with previous studies on *Bertholletia* fruit production (e.g., [32, 87, 90, 92, 101–102]), tree size [crown diameter (P≤0.0001)] is an important contributing factor to total nut weight per tree. However, DBH was not an important variable in determining total nut weight per tree. Although we would have expected a stronger relationship between DBH and total nut weight, we know that the relationship between DBH and crown diameter in our site is not completely linear (see Fig 5). Observing the spread of the data, one can see that while the largest crowns were consistently the largest producers, the same cannot be said of the DBH covariate, which has a much weaker relationship with nut weight in our study sites (see Fig 8).

Of the four different crown position categories, only the intermediate one was a good predictor for total nut weight. While we would have expected the “suppressed” label to be a stronger predictor than “intermediate”, this trend may be an artifact of the subjective nature of the categories used in recording data, particularly among these two categories. While it is true that very few trees were classified in these two categories (most of our trees were “dominant” or “codominant”), thus creating an unbalanced data set, we submit that this issue is typical of observational data sets. The generalized linear model function, as fitted by the R package lme4, is particularly effective in dealing with this issue.

Presence of lianas (P≤0.01) was also an important variable in explaining fruit production. Studies from neighboring Acre, Brazil demonstrate strong evidence supporting the removal of lianas in order to improve fruit production [32, 92]. All five of the concessionaires associated with this study claim to clean their Brazil nut trees from lianas. Supporting that assertion, of the 499 Brazil nut individuals included in this study, only 17%, or 86 trees, had lianas present. Although we did not see an influence of logging damage (or for that matter, any type of damage) to the trees in this study, it is strongly recommended that lianas also be removed in order to avoid collateral damage to the Brazil nut trees during logging operations (see [103]).

## Conclusions

The economic, ecological, and cultural importance of Brazil nut in the tri-frontier MAP region cannot be overstated. Yet, the diverse values associated with this keystone species cannot deter smallholders from selling their timber [20], despite potential risks that inevitably accompany the removal of large trees from the forest. All five concession holders with whom we worked during this study do not believe that logging has a long-term negative impact on Brazil nut production. During our initial meetings, many concessionaires expressed opinions to the contrary, but they nonetheless believed that the potential income generated from timber sales justified the risk to their NTFP resource. The belief that logging can be managed sustainably and the consistent cash flow provided by timber suggest that timber will maintain its status as a key component of regional local livelihoods.

One constraint to our study was conducting the research in sites that have been consistently logged for timber for at least three decades. But we posit that exploited and degraded forests are increasingly the norm in the tropics [104], and that our design (i.e., distance to logging as one of the principle explanatory variables) was the best compromise to account for possible changes in production levels barring the presence of an undisturbed forest site (see [45, 93]). And while we do concede that it is normally be ideal to work with a long-term data set, we have a large sample size across an extensive geographical area, and results (with the exception of the site where logging intensity was higher) were consistent across the different concessions during both years of the study.

Given the results of our study, we suggest that under certain conditions, it is possible to manage Brazil nut in concert with timber in this region as long as logging crews remain at least 100 m from reproductive Brazil nut trees, providing the basis for proximal compatibility of the two livelihood strategies. Instituting a buffer zone (similar to those mandated for riparian areas in forests managed for timber) around individual Brazil nut trees or groves would provide an added layer of protection from both primary (e.g., direct damage to tree from harvesting activities) and secondary (e.g., windfall as a result of logging) impacts. Already in Madre de Dios, such organizations as the Association for the Conservation of the Amazon Basin (ACCA) and the Federation of Brazil Nut Producers of Madre de Dios (FEPROCAMD) engage in concession-scale inventories for reproductive Brazil nut trees. Yet calculating distances of Brazil nut trees from commercial timber trees (in order to facilitate and plan logging operations) is not yet mandated by the government. In contrast, in neighboring Pando, Bolivia, local NGOs and the Forest and Land Authority are helping communities to create detailed inventories of Brazil nut tree populations and their proximity to commercial timber species via the “Integrated Forest Management Plans” [26].

Low logging intensities (1–2 trees ha^-1^) should always be observed, but especially when it is necessary to enter the primary grove. Indeed, we recognize that it is unlikely that Brazil nut concessionaires will consistently avoid areas of high concentration of Brazil nut trees when harvesting timber, especially as the demand for timber increases and as stocks deplete within the concessions. As a comparison, logging intensity levels found in most of these sites is considerably lower (approximately 5–10 m^3^ ha^-1^) than what is found in other Brazil nut-rich areas of the Amazon (15–35 m^3^ ha^-1^; [45, 105–106]). Even though most smallholder forest income in Madre de Dios is still derived from Brazil nuts [107], market demand for timber is increasing across the Amazon Basin [108], thus limiting the ability of governments to propose and enforce reduced logging intensities, a practice which would diminish short-term economic return. Risk of damage to the forest is also increased when third-party loggers become involved, a common practice in Madre de Dios and the rest of the Amazon (see [109–111]).

Perhaps one of the strongest incentives for forest smallholders (and even larger logging operations) to keep Brazil nut trees is that the species in question is protected in Bolivia, Brazil, and Peru. While this restriction may or may not be effective in terms of protecting the natural resource base in cases of complete deforestation (see [112]), it may be serve as a safeguard for Brazil nut groves during selective logging operations. There has also been a strong push for the certification of both timber and Brazil nut in recent years (see [54, 113–114]). Despite varying degrees of success of that effort, there is still potential for the inclusion of multiple use forest management mandates in certification criteria, potentially boosting the value of products originating from these smallholder-managed forests.

The southwestern Amazon region provides an ideal landscape in which to study multiple-use forestry, given the important roles that both Brazil nut and timber play in the regional economy. Considering the results we have outlined in this paper, we contend that smallholders should precede with caution in regards to timber extraction in Brazil nut concessions. Our results further provide a platform for discussion on the recently-approved Peruvian Forestry Law, which states (in its article 57) that timber may be extracted from concessions destined to NTFP extraction as long as the NTFP resource is maintained [115]. Yet the ability of Brazil nut concessionaires to adhere to this regulation is limited by the lack of data in our study region. While a logging permit is required, there is no procedure in place for generating timber inventory data across a particular Brazil nut concession for sound, multiple use planning. This is a common problem for smallholders not only in Madre de Dios but across the MAP region [18]. Ecological studies that focus on these smallholder systems are critical for generating information that could allow forest managers to modify and improve their livelihood strategies. Yet, there are still many factors missing, including technical and financial support for creating management plans, widespread implementation of reduced-impact logging methods, control of illegal extraction, incorporation of local knowledge and preferences into government-mandated forest plans, and promotion of dialogue between different stakeholders [3, 26, 40, 116]. If complementary research efforts can be coordinated with NGOs, regional governments, and smallholders, information can be generated to develop new management strategies that will help forest managers pursue two profitable livelihood strategies simultaneously.

.
